# Assessment of Citrinin in Spices and Infant Cereals Using Immunoaffinity Column Clean-Up with HPLC-Fluorescence Detection

**DOI:** 10.3390/toxins13100715

**Published:** 2021-10-10

**Authors:** Christopher Mair, Michael Norris, Carol Donnelly, Dave Leeman, Phyllis Brown, Elaine Marley, Claire Milligan, Naomi Mackay

**Affiliations:** R-Biopharm Rhone Ltd., Block 10, Todd Campus, West of Scotland Science Park, Acre Rd., Glasgow G20 OXA, UK; carol@r-biopharmrhone.com (C.D.); dave@r-biopharmrhone.com (D.L.); phyllis@r-biopharmrhone.com (P.B.); elaine@r-biopharmrhone.com (E.M.); claire@r-biopharm.com (C.M.); naomi@r-biopharmrhone.com (N.M.)

**Keywords:** mycotoxins, citrinin, immunoaffinity, spices, infant food, HPLC-fluorescence

## Abstract

Historically, the analysis of citrinin has mainly been performed on cereals such as red yeast rice; however, in recent years, more complex and abnormal commodities such as spices and infant foods are becoming more widely assessed. The aim of this study was to develop and validate clean-up methods for spices and cereal-based infant foods using a citrinin immunoaffinity column before HPLC analysis with fluorescence detection. Each method developed was validated with a representative matrix, spiked at various citrinin concentrations, based around European Union (EU) regulations set for ochratoxin A (OTA), with recoveries >80% and % RSD < 9% in all cases. The limit of detection (LOD) and the limit of quantification (LOQ) were established at 1 and 3 µg/kg for spices and 0.1 and 0.25 µg/kg for infant cereals, respectively. These methods were then tested across a variety of spices and infant food products to establish efficacy with high recoveries >75% and % RSD < 5% across all matrices assessed. Therefore, these methods proved suitable for providing effective clean-up of spices and infant cereals, enabling reliable quantification of citrinin detected. Samples such as nutmeg and infant multigrain porridge had higher levels of citrinin contamination than anticipated, indicating that citrinin could be a concern for public health. This highlighted the need for close monitoring of citrinin contamination in these commodities, which may become regulated in the future.

## 1. Introduction

Citrinin (CIT) is a secondary fungal metabolite produced by several species of the genera *Aspergillus*, *Penicillium*, and *Monascus* [1]. It is known to be found in variety of commodities such as maize, oats, rice, and rye, generally formed by fungi after harvest when stored in high-humidity environments [1,2]. CIT is known for causing nephrotoxicity (toxicity in the kidneys) and has a tolerable daily intake (TDI) value of 0.2 μg/kg body weight [1,3]. It is known to be rapidly absorbed by the liver and kidney, with a recent CIT toxicokinetic study in humans showing that only 40% was excreted in urine, indicating that its absorption was greater than 40% [1,4].

CIT is often found to co-occur with ochratoxin A (OTA), showing similar structural and toxicological properties, which is a cause for concern for both human and animal health [5]. It is thought that CIT is less potent than OTA, yet has been found at considerably higher levels in food and feed [5,6]. Thus, with the combined toxic effects of OTA, it is important that CIT exposure in humas is assessed across a variety of commodities in which OTA is likely to occur. However, limited occurrence data for CIT in food matrices are currently available to establish reliable dietary exposure assessments, with only a few preliminary studies having taken place such as in Belgium in 2020 (Meerpoel, Vidal) [5,7].

The main focus of citrinin exposure has been from red yeast rice, which is intentionally molded to make various dietary supplements and food colouring agents, primarily in Asia [8,9] and is currently the only matrix type which is included in EU legislation (Regulation (EC) 1881/2006) [10]. However, in recent years, studies have been carried out assessing CIT occurrence in more complex and abnormal food matrices such as spices and infant food, the focus of this article, where moderate to high levels of CIT contamination have been found, particularly in spices. For instance, a study carried out in 2015 by Jeswel and Kumar on the natural incidence of CIT in local Indian spices reported CIT contamination in a variety of spices, with red chilli, black pepper and dry ginger containing the highest CIT concentrations [11]. CIT contamination was most frequently detected (47.2%) in red chilli, with dry ginger showing the highest average concentration at 85.1 µg/kg [6,11]. The report highlights the need for care in the storage of spices upon processing, handling and transportation to minimize the development of mycotoxin growth [11]. Similarly, a dietary exposure assessment carried out in Belgium by Meerpoel and Vidal in 2020 characterised the risk of CIT and OTA in a variety of foodstuffs, including herbs and spices [7]. It was found that 50% of the herbs and spices assessed were contaminated with CIT, with the maximum level found at 4.5 µg/kg [7]. It was determined that for herbs and spices, the daily intake of CIT by average consumers could reach upper bound concentrations of approximately 0.140 and 0.0556 ng/kg body weight per day in 3–9 year olds and 18–64 year olds, respectively [7]. This study therefore addressed the need for further research concerning CIT and OTA combined exposure in spices, particularly in young children, where higher CIT exposure was observed.

Studies regarding infant foods are more infrequent, with CIT contamination in infant and young children mostly being studied as part of co-exposure assessments with a variety of mycotoxins. For instance, a study conducted in 2018 by Ojuri and Ezekiel assessed mycotoxin co-exposure in infants and young children consuming household and industrially processed complementary foods, including infant formula and family cereals, across two Nigerian states [12]. Exposure levels of citrinin were calculated to be in the range of 0.0–0.5 and 0.0–13.6 µg/kg body weight per day for infant formula and family cereal, respectively, with higher CIT contamination found in household products [12]. The exposure estimates were considered high, particularly with maize-based products, resulting in risk management advice being made available to relevant stakeholders for mycotoxin control such as good agricultural practices and food safety awareness [12]. In addition to this, a study carried out by Ali and Degen in 2020 detailed biological monitoring of OTA, CIT and their metabolites in urine samples of infants and young children in Bangladesh [13]. It was found that CIT biomarkers were detectable in 54.9% of urine samples, with the total CIT biomarker concentration notably higher in children (2.16 ng/mL) than in infant (0.70 ng/mL) urine. The provisional daily intake for CIT was calculated and exceeded the value currently set (0.2 µg/kg body weight) in 23.3% and 11.9% of children and infants, respectively, where CIT biomarker concentrations were found to be higher in young children than in Bangladeshi adults in the summer [13]. These studies demonstrate the necessity of regular surveillance of food consumed by infants and young children to identify major sources of CIT intake [13], highlighting the importance of developing robust and reliable methods for the extraction of CIT in infant foods.

As of 2019, changes have been made to the EU legislation surrounding food supplements containing red yeast rice, with Section 2.8.1 of the Annex to Regulation (EC) 1881/2006 amended to state that the maximum legislative level for CIT has been decreased significantly from 2000 to 100 µg/kg [10,14]. These levels are broadly similar in China and Japan, with the maximum limits for CIT in red yeast rice set at 50 and 200 µg/kg, respectively [6]. This change can present challenges such as increased background interference in detection methods, particularly if working with complex matrices, and a need for increased sensitivity to reach lower limit of quantification (LOQ) and limit of detection (LOD) requirements. Therefore, better clean-up after initial CIT extraction may be required.

Many clean-up options are available such as liquid–liquid extraction (LLE); solid-phase extraction (SPE) [15]; Quick, Easy, Cheap, Effective, Rugged and Safe (QuEChERS) [16]; and immunoaffinity columns (IAC) [17,18]. For complex matrices such as spices, less specific clean-up methods such as LLE and SPE can suffer from matrix interferences, leading to poor separation of the target analyte and noisy chromatograms, particularly when assessing at trace levels [18]. One method used to overcome matrix effects is through the use of IAC, which uses a specific antibody that binds to the target compound when passed over the affinity sorbent. This is highly selective and can improve the sensitivity for analysis at low detection levels [18].

Quantification of CIT is primarily carried out by either HPLC-fluorescence or LC–MS/MS detection. LC–MS/MS methods have become more popular in recent years; however, the natural fluorescence of CIT provides an attractive substitute, with the sensitivity of HPLC-fluorescence demonstrating comparable results to that of LC–MS/MS [18,19]. This is particularly useful for labs that do not possess an LC–MS/MS instrument due to various issues such as cost or lab space.

Previous work, carried out by R-Biopharm Rhone Ltd., assessed red yeast rice spiked at various levels, including the now lowered maximum limit of 100 µg/kg CIT, with IAC clean-up (EASI-EXTRACT^®^ CITRININ) with HPLC-fluorescence detection as part of a single lab validation with replicate analysis conducted across 3 consecutive days. High % recovery with clear chromatograms, free from interfering compounds, was achieved across all spike levels assessed demonstrating the effective clean-up gained from using IAC [2].

With the lowered maximum levels set for red yeast rice supplements, it is anticipated that strict levels may come into place for more complex or abnormal commodities such as spices and cereal-based infant foods that are now being more widely tested [6,7,11,12,13]. These levels could potentially be similar to the maximum limits set for the associated toxin OTA. For OTA, the current maximum legislative levels set by Regulation (EC) 1881/2006 for spices and cereal-based infant foods are 15 and 0.5 µg/kg, respectively [10]. Therefore, as there is currently no legislation in place for CIT in these matrix types, the commodities in this paper were assessed against EU regulation criteria available for OTA [10,20].

The aim of this study was to develop and validate methods for the extraction of CIT, at low levels, in spices and cereal-based infant foods using IAC clean-up (EASI-EXTRACT^®^ CITRININ, R-Biopharm Rhone Ltd., Glasgow) before analysis with HPLC- fluorescence. The validated methods were then assessed across various spices and infant cereals to assess the natural occurrence of CIT and establish efficacy of each method across these different commodities.

## 2. Results and Discussion

### 2.1. Method Validation Results

For both spices and cereal-based infant food, the cereal extraction method provided in the instructions for use for EASI-EXTRACT^®^ CITRININ IACs underwent slight modifications in order to remove background interference for spices and to increase the sensitivity of CIT in infant food. For spices, this included the addition of 10% Tween 20 in the extraction filtrate dilution and increasing the volume passed through the IAC. For cereal-based infant food, modifications included an increased proportion of sample in extraction, increased dilution of extraction filtrate and increased volume passed through the IAC. These final methods, found in Section 4.3, were then validated.

The spice method was validated with “blank” and spiked chilli powder samples, spiked at 0.5×, 1× and 2× the legislative level (LL) that is set for OTA in spices by EU Regulation (EC) 1881/2006 (7.5, 15 and 30 µg/kg, respectively) as well as at a LOQ of 3 µg/kg (1/5th LL) and a LOD of 1 µg/kg (1/3rd LOQ). Similarly, the cereal-based infant food method was validated with “blank” and spiked infant porridge powder samples, spiked at 0.5×, 1× and 2× the LL that is set for OTA in infant food by EU Regulation (EC) 1881/2006 (0.25, 0.5 and 1 µg/kg, respectively) as well as at a LOQ of 0.25 µg/kg (1/2 LL) and a LOD of 0.1 µg/kg (2/5th LOQ). Each spike level was assessed across three IACs, where the spiked recovery values and precision, expressed as repeatability (% RSD), between replicates were calculated. The validation results are shown in Table 1.

**Figure 1:**
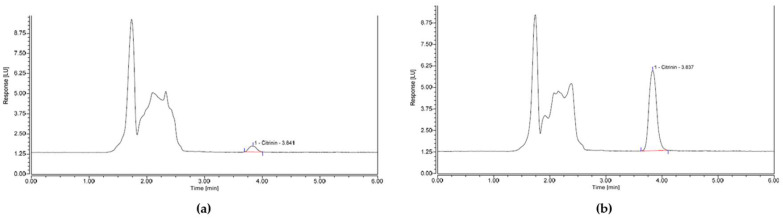
HPLC chromatograms for (**a**) chilli powder spiked with citrinin (CIT) at a limit of detection (LOD) concentration of 1 µg/kg and (**b**) chilli powder spiked with CIT at the proposed LL concentration of 15 µg/kg.

The validation of the spice method was successful, with recovery >80% for all CIT spike levels assessed and with no natural CIT contamination found in the “blank” chilli powder. Variation (% RSD) between triplicate IACs was also below the typical maximum limit of 20%, set by EU Regulation (EC) 401/2006, at <6% across all spike levels. Clean-up quality of the samples was acceptable, with no interfering peaks present. Clean and sharp peaks were observed from low CIT concentrations at a LOD of 1 µg/kg to higher concentrations at a LL of 15 µg/kg, as shown in Figure 1.

**Figure 2:**
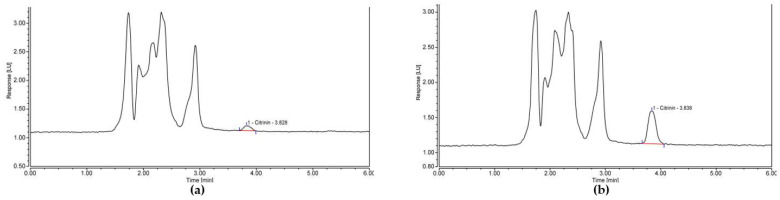
HPLC chromatograms for (**a**) infant porridge spiked with CIT at a LOD concentration of 0.1 µg/kg and (**b**) infant porridge spiked with CIT at the proposed LL concentration of 0.5 µg/kg.

Similarly, the validation of the cereal-based infant food was successful, with recovery between 100 and 110% for all CIT spike levels assessed, with no natural CIT contamination found in the “blank” infant porridge. Variation (% RSD) between triplicate IACs was also below the typical maximum limit of 20% at <9% across all spike levels. Clean-up quality of the samples was acceptable, with no interfering peaks present. Clean and sharp peaks were observed from low CIT concentrations at a LOD of 0.1 µg/kg to higher concentrations at a LL of 0.5 µg/kg, as shown in Figure 2.

The LOQ of an infant food method is usually expected to be approximately 2/5th that of the proposed legislative limit of the appropriate analyte—in this instance, 0.2 µg/kg CIT. The LOQ achieved was slightly higher at 0.25 µg/kg CIT, due to a poorer signal-to-noise (S/N) ratio, below the expected 10, at concentrations lower than this. However, the method used in this study aims for maximum sensitivity by utilising a larger sample weight while still maintaining effective extraction, diluting the extract as little as possible while still considering the solvent tolerance of the IAC, and concentrating the analyte on the column by passing a larger volume through while still maintaining practicality. Therefore, with consideration, this deviation from the typical performance criteria may be deemed acceptable considering the LOQ is system dependent and there is no legislative limit or performance criteria currently in place for CIT in infant food.

### 2.2. Sample Study

The validated spice and cereal-based infant food methods were tested across six different spices and six different infant cereals acquired from various international suppliers to assess the reliability and robustness of the validated methods. Each sample was tested “blank” to assess natural contamination and spiked at the legislative limit that is set for OTA by EU Regulation (EC) 1881/2006 across three IACs, where the spiked recovery values and % RSD between replicates were calculated. The natural contamination in each sample was then corrected based on the recovery achieved for each sample assessed. The results from this study are shown in Table 2 and Table 3.

Upon assessment of the various spices with the validated spice method, “blank” samples revealed small concentrations of CIT in cinnamon and ginger, but far below the proposed legislation of 15 µg/kg. However, the nutmeg sample contained a significantly higher natural level of CIT (44.3 µg/kg) which indicates that natural occurrence of CIT in spices is of concern.

**Figure 3:**
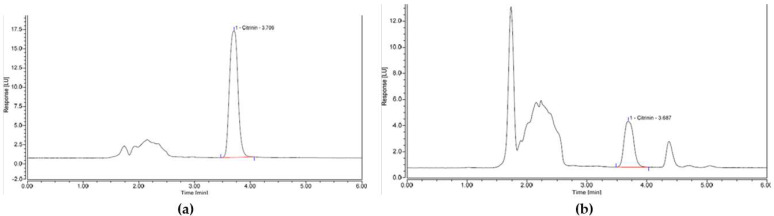
HPLC chromatograms for (**a**) nutmeg spiked with CIT at the proposed LL concentration of 15 µg/kg and (**b**) turmeric spiked with CIT at the proposed LL concentration of 15 µg/kg.

The recovery of CIT obtained from all spice samples was within the acceptable range of 70–110% set by the EU Regulation (EC 401/2006) for similar mycotoxins and variation between triplicate IACs was also well below the typical maximum limit of 20%. Clean-up quality was generally good as four out of six samples did not have any interferences or obstructing peaks. Chromatograms obtained from the turmeric and cinnamon sample contained an additional peak however, the effect of the additional peak had no significant impact on the accuracy of the analysis. Example chromatograms of spiked spice samples assessed in this study are shown in Figure 3.

Upon assessment of the various infant cereals with the validated infant cereal method, blank samples revealed a significant contamination of CIT in the multigrain porridge (9.08 µg/kg), significantly higher than the equivalent maximum concentration of 0.5 µg/kg in place for OTA by EU regulations (EC 1881). This indicates that monitoring these commodities is of importance in the interest of public health, particularly when fed to babies and young children.

**Figure 4:**
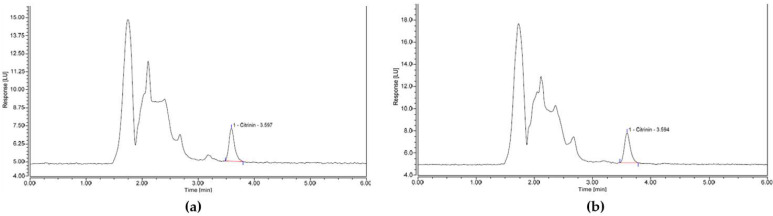
HPLC chromatograms for (**a**) infant oat cereal spiked with CIT at the proposed LL concentration of 0.5 µg/kg and (**b**) multigrain porridge spiked with CIT at the proposed LL concentration of 0.5 µg/kg.

With the exemption of spelt-based infant cereal, recovery of CIT from all spiked infant cereal samples was within the acceptable range of 50–120% set for OTA at levels < 1 µg/kg with % RSD < 5% for all infant cereals. However, as recovery was only 6.5% over the maximum limit for spelt cereal and no applicable performance criteria for CIT analysis have been published, the method could be deemed as suitable for the analysis of CIT in cereal-based infant foods. Clean-up quality for all samples was good as no interfering peaks were observed near the retention time of citrinin (3.6 min) in any chromatograms, with other unresolved matrix specific peaks eluting before the CIT retention time (1.5 to 3 min). Example chromatograms of spiked infant cereal samples assessed in this study are shown in Figure 4.

### 2.3. Results Comparison

As discussed, there are a variety of different types of clean-up and detection methods for the assessment of CIT, with only a small selection detailing the assesment of CIT in spices and infant food. For instance a study carried out by Yogendrarajah in 2013 demonstrated the use of a QuEChERS clean-up method with LC–MS/MS detection to assess multiple toxins in a variety of spices [21]—namely, black pepper, white pepper and red chilli. The LOQ results for CIT were high and ranged from 65 to 146 µg/kg for these samples. The spices were spiked with CIT in the range of 80–400 µg/kg, with recoveries ranging from 93 to 106% with a repeatability (% RSDr) between 1 and 11% [21]. Moreover, a study was conducted by Meerpoel in 2018 which similarly made use of LC–MS/MS detection for the assessment of CIT and OTA in various foodstuffs including spices (nutmeg) and baby milk powder [22]. Nutmeg was validated in the spike range of 1–10 µg/kg, with recoveries ranging from 80 to 96% with a repeatability (% RSDr) between 12.5 and 17.6%. LOD and LOQ results were 0.8 and 1.6 µg/kg, respectively. Baby milk powder was validated in the spike range of 0.5–10 µg/kg, with recoveries ranging from 70 to 99% with a repeatability (% RSDr) between 8 and 21%. LOD and LOQ results were 0.3 and 0.5 µg/kg, respectively [22].

Therefore, if we were to compare the results from this study, the spice recovery results obtained upon validation and in the spice sample study were between 81.8 and 85.4% and 77.2 and 99.5%, respectively, with % RSD remaining below 3%. When comparing to the 2013 and 2018 study results, the validation recovery range is narrower across varied spike levels than that observed in both papers mentioned (93–106% in 2013 study, 80–96% in 2018 study). The % recovery results for the sample study are slightly more varied than in the 2013 and 2018 studies. This could be in part be due to the lower spike levels assessed as well as the variety of spices that were tested which have different interactions with the extraction procedure and IAC, leading to slight variations between different spices. However, for each spice assessed, the % RSD remained significantly lower, <3%, when compared to both studies, i.e., 12.5–17.6% for nutmeg in the 2018 study, indicating that the results were precise, with little variation when assessing the IAC with the same spice. For this study, the LOD and the LOQ achieved were 1 and 3 µg/kg, respectively. LOD and LOQ results were also comparable with the Meerpoel and Yogendrarajah studies, where similar or lower LOD and LOQ values were established, with only slightly higher levels than the LOD and the LOQ of 0.8 and 1.6 µg/kg, respectively, attained in the 2018 study. This indicates the level of sensitivity that can be achieved with IAC clean-up.

For infant cereals, the recovery results obtained upon validation and in the infant cereal sample study were between 102.2 and 108.4% and 75.4 and 126.5%, respectively, with % RSD remaining below 9%. Similar to spices, the infant food validation recovery range is narrower across varied spike levels than that observed in the 2018 study (70–99%). For the sample study, the % recovery results are more varied. This could be due to the fact that a variety of infant cereals were tested which have different interactions with the extraction procedure and IAC. However, for each infant cereal assessed, the % RSD remained significantly lower, <9%, when compared to the 2018 study (8–21%), indicating that the results were precise with little variation when assessing the IAC with those same infant cereal. LOD and LOQ levels for infant cereals in this study were 0.1 and 0.25 µg/kg, respectively. LOD and LOQ results were also comparable, with lower LOD and LOQ levels than observed in the 2018 study (LOD of 0.3 µg/kg and LOQ of 0.5 µg/kg), indicating the increased sensitivity that can be achieved with IAC clean-up. It should be noted, however, that sensitivity can be dependent on the specifications of the detection method used, i.e., HPLC—fluorescence vs. LC–MS/MS.

## 3. Conclusions

To conclude, this paper has reported the application of commercially available immunoaffinity columns in conjunction with HPLC-FLD detection for the clean-up of citrinin in a variety of spices and cereal-based infant foods which are now being more frequently assessed. Clear purified extracts were obtained from clean-up with EASI-EXTRACT^®^ CITRININ, allowing for accurate determination of citrinin, with no interference from any closely eluting compounds. This provided selective and specific identification and quantification of citrinin from low to high concentrations. Percent recovery and % RSD met the requirements set for similar analytes (OTA) by EU Regulations across all matrices assessed in this study with the spice and infant cereal methods developed. Matrices such as nutmeg and infant multigrain porridge contained considerably higher levels of citrinin than expected, making it clear that citrinin content in spices and cereal-based infant foods is of concern for public health, particularly in babies and young children, and should be closely monitored. With this and the recent decrease in the maximum legislative limit set by the EU for red yeast rice, it is anticipated that legislation may be put in place for spices and cereal-based infant products at low to moderate maximum legislative levels, highlighting the need for reliable and sensitive clean-up methods as described in this study.

## 4. Materials and Methods

### 4.1. Materials and Chemicals

#### 4.1.1. Standards and Immunoaffinity Columns

Citrinin powder (>98% pure) was acquired from Sigma Aldrich, of which 5 mg was reconstituted in 5 mL of methanol to give a concentration of 1 mg/mL. This was then diluted further with methanol to make 1000 and 100 ng/mL CIT working standards. EASI-EXTRACT^®^ CITRININ immunoaffinity columns from R-Biopharm Rhone Ltd. (Glasgow, United Kingdom) were used for sample clean-up after extraction.

#### 4.1.2. Reagent Preparation

The 10 mM phosphoric acid solution was prepared by dilution of 1.368 mL 85% phosphoric acid to 2 L of deionized water and the pH was adjusted to 2.5 with 5 M sodium hydroxide. For 10 mM phosphoric acid (pH = 7.4), 500 mL of 10 mM phosphoric acid was adjusted to pH = 7.4 with 5 M sodium hydroxide. 0.25 g of Tween 20 was added to 250 mL of 10 mM phosphoric acid (pH = 7.4) to obtain 0.1% Tween 20 in 10 mM phosphoric acid (pH = 7.4). For the PBS solution, 1 PBS tablet was dissolved per 100 mL water to give a solution containing 8.0 g/L sodium chloride, 0.2 g/L potassium chloride, 1.15 g/L di-sodium hydrogen phosphate, and 0.2 g/L potassium dihydrogen phosphate.

Methanol and acetonitrile were sourced from Thermo Fisher (Loughborough, United Kingdom) and deionized water was prepared using Millipore Elix, followed by Milli-Q Academic (Watford, UK). Phosphoric acid 85% and Tween 20 were obtained from Sigma Aldrich and PBS tablets were obtained from R-Biopharm Rhone Ltd.

### 4.2. Samples and Spiking Procedure

A variety of spices and cereal-based infant food samples were obtained from both local and international sources. Control spice and infant food samples were chosen for method validation at various spike levels. A small sample study was then carried out with 6 different spice and infant food samples from international suppliers to assess CIT content. For spices and cereal-based infant foods, each sample was spiked with the appropriate volume of CIT working solution in methanol. The sample was then left for at least 30 min to allow the methanol to evaporate. A “blank” sample was assessed alongside each spiked sample to assess natural CIT contamination, accounted for in recovery calculations.

### 4.3. Extraction Methods

#### 4.3.1. Spices

A variety of spices were assessed for CIT by first weighing 25 g of ground sample into a 1 L capacity, solvent-resistant blender jar. A volume of 100 mL of 75% methanol was then added and the mixture was blended at high speed for 2 min. The extraction solution was passed through Whatman No. 113 filter paper before diluting 20 mL of filtrate in 80 mL of 10% Tween 20 in PBS. The diluted solution was filtered through glass microfibre (GMF) paper before passing 20 mL through an EASI-EXTRACT^®^ CITRININ IAC at 2 mL/min. The column was washed with 10 mL of 0.1% Tween 20 in 10 mM phosphoric acid (pH = 7.4) followed by 10 mL of 10 mM phosphoric acid (pH = 7.4) at 5 mL/min. The toxin was eluted into an amber glass vial with 1 mL of methanol followed by 1 mL of water to give a 2 mL volume. A volume of 100 µL was then injected onto the HPLC system.

#### 4.3.2. Cereal-Based Infant Foods

A variety of cereal-based infant foods were assessed for CIT by first weighing 60 g of ground sample into a 1 L capacity, solvent-resistant blender jar. A volume of 200 mL of 75% methanol was then added and blended at a low speed for 2 min. The extraction solution was passed through Whatman No. 113 filter paper before diluting 30 mL of filtrate in 120 mL of PBS. The diluted solution was filtered through GMF paper before passing 40 mL through an EASI-EXTRACT^®^ CITRININ IAC at 2 mL/min. The column was washed with 10 mL of 0.1% Tween 20 in 10 mM phosphoric acid (pH = 7.4) followed by 10 mL of 10 mM phosphoric acid (pH = 7.4) at 5 mL/min. The toxin was eluted into an amber glass vial with 1 mL of methanol followed by 1 mL of water to give a 2 mL volume. A volume of 100 µL was then injected onto the HPLC system.

### 4.4. Calibration Standards, Recovery, LOD and LOQ

Linearity was evaluated using a bracketed calibration series prepared in 50% methanol by serial dilution. The concentration ranges used for this study were between 0.0375 and 30 ng/mL CIT. Calibration curves were constructed by plotting the peak areas (y) versus the concentration of analytes (x). The % recovery was calculated from the ratio of the predicted value obtained from the calibration curve divided by the actual/theoretical value times 100.

LODs and LOQs were determined by measuring the average signal-to-noise ratio in samples spiked at appropriate LOD and LOQ concentrations and taking the LOD to be equal to 3-fold the noise level and the LOQ to be equal to 10-fold the noise level. The LOD was prepared by pooling “blank” final eluates (post IAC) and spiking at the equivalent LOD concentration. The LOQ was prepared by spiking the appropriate LOQ concentration directly onto the sample before extraction.

### 4.5. HPLC Conditions

HPLC analysis was carried out using an Agilent 1260 Infinity II HPLC system with a florescence detector set at λex = 330 nm and λem = 500 nm. A 150 × 4.6 mm, 3 μm Hypersil GOLD LC column (Thermo Fisher Scientific) was used isocratically with a (50/50 *v/v*) acetonitrile −10 mM phosphoric acid (pH = 2.5) mobile phase at a flow rate of 1 mL/min and a column oven temperature of 40 °C. Under these conditions, citrinin elutes with a retention time of approximately 3.6 min and a total run time of 6.0 min.

## Figures and Tables

**Table 1:** Method validation results.

Matrix	CIT Spike Level (µg/kg)	Calculated CIT Concentration (µg/kg)	Recovery (%)	Precision RSD (%)
Chilli	3	2.56	85.4	2.56
	7.5	6.40	85.3	0.58
	15	12.35	82.3	0.36
	30	24.54	81.8	1.79
Infant porridge	0.1	0.106	105.6	8.31
	0.25	0.26	102.2	4.47
	0.5	0.52	104.8	1.78
	1	1.08	108.4	0.93

**Table 2:** Spices sample study results.

Matrix	Calculated “Blank” CIT Corrected Concentration (µg/kg)	Calculated Spiked CIT Concentration (µg/kg)	Recovery (%)	Precision RSD (%)
Cinnamon	0.43	11.58	77.2	1.48
Nutmeg	44.32	14.93	99.5	2.86
Ginger	0.55	12.87	85.8	0.02
Paprika	0.00	12.38	82.5	0.28
Turmeric	0.00	13.06	87.1	0.89
Chilli	0.00	13.16	87.7	0.58

**Table 3:** Cereal-based infant food sample study results.

Matrix	Calculated “Blank” CIT Corrected Concentration (µg/kg)	Calculated Spiked CIT Concentration (µg/kg)	Recovery (%)	Precision RSD (%)
Oatmeal, Quinoa and Millet Mix	0.00	0.39	79.8	4.82
Semolina	0.00	0.42	83.9	2.35
Baby Rice	0.00	0.39	77.5	1.44
Oat Cereal	0.00	0.37	75.4	2.03
Spelt Cereal	0.00	0.63	126.5	0.55
Multigrain Porridge	9.08	0.42	83.9	4.38

## Data Availability

The data presented in this study are available on request from the corresponding authors. The data are not publicly available due to company privacy.
